# The Learning Curve in Robotic HPB Surgery: Myth, Metrics, and Misinterpretations

**DOI:** 10.3390/jcm15208043

**Published:** 2026-10-16

**Authors:** Silvio Caringi, Antonella Delvecchio, Annachiara Casella, Valentina Ferraro, Matteo Stasi, Nunzio Tralli, Tommaso Maria Manzia, Riccardo Inchingolo, Michele Tedeschi, Riccardo Memeo

**Affiliations:** 1 Unit of Hepato-Biliary and Pancreatic Surgery, “F. Miulli” General Hospital, 70021 Acquaviva delle Fonti, Italy; 2 Department of Medicine and Surgery, LUM University, 70010 Casamassima, Italy; 3 Transplant and HPB Unit, Department of Surgery Sciences, University of Rome Tor Vergata, 00133 Rome, Italy; 4 Unit of Interventional Radiology, “F. Miulli” General Hospital, 70021 Acquaviva delle Fonti, Italy

**Keywords:** robotic surgery, hepatopancreatobiliary surgery, learning curve, surgical training, competency-based assessment

## Abstract

**Background**: The evolution of robotic surgery has widened the indications for minimally invasive hepatopancreatobiliary (HPB) surgery, making possible the execution of more complicated cases. Within this context, the learning curve concept is now an essential factor when assessing surgical training and expertise, as well as patient safety within a robotic HPB program. Nevertheless, existing methodologies often make use of procedure volume and surrogate intraoperative measures, which may be an insufficient way to define surgical competence. **Methods**: This narrative review examines the learning curve concept in robotic HPB surgery, focusing on existing performance metrics, methodological limitations, procedural complexity, risk adjustment, and the influence of team-related and institutional factors. **Results**: The time of operation, the estimated blood loss, the rate of conversion, and postoperative morbidity are frequently applied to assess learning curves. Nevertheless, these metrics are affected by the criteria of patient selection, the level of complexity of the procedure, and some other factors like institutional characteristics and experience of the team performing the procedure. As a result, one single value or an inflection point might fail to adequately capture the concept of surgical competence. The concepts of competency, proficiency, and mastery are distinct stages of learning, whereas the speed of learning for such skills as technical proficiency, clinical outcomes, oncological sufficiency, and intraoperative decision-making might be different. **Conclusions**: The learning curve in robotic HPB surgery should be viewed as a multi-dimensional and complex process and not a straightforward correlation between the number of cases and the results. The future assessment of the learning curve needs to consider different domains, including technical, clinical, oncological, cognitive, and team-related aspects, taking into account the level of complexity and institutional context of each case.

## 1. Introduction

Robotic surgery has been one of the primary impetuses that have pushed minimally invasive hepatopancreatobiliary (HPB) surgery into the next level during the past 10 years. Thanks to the technical benefits provided by robotic platforms, including the availability of three-dimensional visualization, wristed instruments, tremor filtration, and ergonomic improvements, the progressive increase of the indications for minimally invasive liver surgery (MILS) has included more complex interventions, such as major hepatectomies, posterior/superior segment resection, and multiple or simultaneous operations for colorectal liver metastases [1,2].

Published learning-curve estimates are procedure-, endpoint-, and context-specific. For example, Rahimli et al. described successive phases across 100 robotic liver resections, with approximately 65 cases associated with their later performance phase [3]. In contrast, Boone et al. identified improvements in blood loss after approximately 20 robotic pancreatoduodenectomies and pancreatic fistula outcomes after approximately 40 procedures [4]. These figures are descriptive findings from individual series, not interchangeable certification thresholds.

Certainly, the present literature tends to utilize predominantly surrogate intraoperative measurements such as the operative time, estimated blood loss, and conversion rates to characterize the learning process. Although easy to collect and quantify, these measures give merely a partial picture of surgical performance, since the operative time decreases with gaining proficiency in using the robotic system but does not necessarily reflect the quality of the dissection, the oncologic adequacy, and the intraoperative decisions made. The amount of estimated blood loss depends on several factors, not limited to the surgical skills, and including the tumor’s features, the parenchyma condition, and the anesthetic management [5].

Moreover, the increasing complexity of the procedures performed makes the traditional approach to the learning curve as a linear one-dimensional phenomenon invalid. In today’s reality, surgical performance is multi-faceted and involves not only the personal abilities of the surgeon but the cooperation between the surgeon and the members of the team, perioperative patient management, the infrastructure of the hospital, and approaches to case selection. The evidence obtained in recent years indicates that the learning process in robotic liver surgery takes place at several levels, such as the personal level, the team level, and the organizational level [6].

One more crucial limitation is the inability of these studies to consider the heterogeneity in case-mix and complexity of the procedures performed. It is impossible to compare the learning curve of minor anterolateral resections with that of major hepatectomies and resections of posterosuperior segments. It has been shown that the results of complexity-adjusted analysis reveal considerable difference in the time and shape of the learning curve between risk-stratified groups, thus pointing to a possible bias inherent to non-adjusted analysis [7].

These points highlight the necessity to rethink the understanding of the learning curve concept in robotic HPB surgery. Oversimplification in the use of metrics and case-number thresholds may distort the real picture of surgical learning process and draw incorrect conclusions on training, credentialing, and practice.

The objective of this narrative review is to critically evaluate the learning curve in robotic HPB surgery. We are going to examine the concepts and metrics used and methodological limitations of the current research, as well as new perspectives based on the multidimensional approach to surgical learning.

## 2. Methods and Scope of the Narrative Review

This narrative review examines the available literature on learning curves in robotic hepatopancreatobiliary (HPB) surgery, with particular emphasis on performance metrics, methodological limitations, procedural complexity, and individual, team-related, and institutional factors. Relevant publications were identified through a narrative literature search and examination of reference lists of pertinent articles. PubMed-indexed publications and publisher websites were consulted using combinations of terms, including “robotic”, “liver resection”, “hepatectomy”, “pancreatoduodenectomy”, “distal pancreatectomy”, “learning curve”, “CUSUM”, “competency”, and “difficulty score”. Publications available up to August 2026 were considered. Particular attention was given to procedure-specific clinical studies, methodological investigations, and publications addressing surgical education and competency assessment. Studies were selected according to their relevance to the objectives of the review, without a formal systematic screening or quality assessment protocol. Accordingly, this review does not aim to provide an exhaustive synthesis of all available evidence, and its findings should be interpreted within the methodological limitations inherent to a narrative review.

## 3. What Do We Mean by “Learning Curve”?

“Learning curve” has become a popular phrase in the surgical literature as a way of explaining how experience and performance progress over time. Nevertheless, in the field of robotic HPB surgery, it still lacks definition and suffers from misconceptions. Traditionally, the learning curve has been explained as the number of operations needed to reach a plateau of some kind of performance, usually operative time. This explanation provides simplicity and quantifiability, but at the same time does not fully reflect the complexity of surgical skills development and can create a superficial perception of the learning process [8,9].

It is important to differentiate between competency, proficiency, and mastery, which denote different levels of surgical development yet are sometimes used interchangeably. Competency denotes the skill to execute surgery safely under regular circumstances with favorable results. Proficiency is an even higher degree of performance marked by reliability and efficiency in a wider array of operations. Mastery includes the possession of high-level technical skills, adaptation to difficult circumstances, and the ability to handle surgical challenges while remaining close to optimal outcomes. There is evidence from extensive series of robotic liver resection surgeries that not only do these levels differ but appear at different periods with early competency reached rather quickly after a certain number of cases, and mastery being attained after much more extensive experience [10,11].

Progression of learning according to the learning curve does not follow any linearity and homogeneity but depends on a multitude of factors, among which previous experience in open and laparoscopic surgeries, minimally invasive surgical skills, case selection approaches, and institutional support. Surgeons with existing skills in laparoscopic surgeries may exhibit an advanced speed of early learning in robotic surgery, especially regarding their effectiveness in performing operations; however, the speed might be compromised with the increase of procedure difficulty [6].

Also, the classical concept of one learning curve that shows the learning progress in its totality does not fully embrace the diversity of surgical performance. The aspects, such as operative efficiency, technical proficiency, complications, and oncological margin adequacy, can develop independently from each other and at different speeds. So, the reduction of operative duration might occur before the stable rate of complications or oncological adequacy is achieved. Thus, the multidimensional approach to surgical learning should be considered [7,12].

Accordingly, learning in robotic HPB surgery cannot be reduced to a one-to-one correlation between case volume and competence. Competency, proficiency, and mastery are useful conceptual labels, but no universally validated, procedure-independent operational thresholds define these stages.

## 4. Procedure-Specific Scope and Transferability

The strongest examples assembled here concern robotic liver resection and robotic pancreatic procedures; evidence specific to robotic biliary reconstruction is less developed. Pancreatoduodenectomy entails anastomotic reconstruction and outcomes such as clinically relevant postoperative pancreatic fistula, delayed gastric emptying, and post-pancreatectomy hemorrhage; distal pancreatectomy has a different technical profile. Boone et al. evaluated 200 robotic pancreatoduodenectomies [4], while Shakir et al. analyzed 100 robotic distal pancreatectomies [13]. The latter study identified operative-time reductions after approximately 20 and 40 cases and lower readmission after 40 cases [13]. These observations support a procedure-specific approach rather than transferring liver thresholds to pancreas or biliary surgery.

## 5. Metrics Used to Assess the Learning Curve

In the assessment of the learning curve for robotic HPB surgery, the quantifiable parameters have been the main means used in the past, and these include both intraoperative and early postoperative results. The commonest among these parameters is operative time, which has been favored because of its objectivity and easy measurability. The decreasing trend of the operating time in each case is usually associated with increasing efficiency of the surgeon and familiarity with the procedure. But although operative time captures early changes in the efficiency of work and coordination in the process, it may not be a measure of surgical proficiency [10,14].

Apart from operative time, other common variables that are usually reported include EBL, conversion to open surgery, and morbidity after surgery. Although such parameters are clinically relevant, they are subject to many confounding factors other than the skills of the surgeon performing the operation. In the case of EBL, for instance, there could be variations due to tumor location, vascular involvement, and the state of the liver parenchyma. On the other hand, conversion rates depend very much on case selection, as well as on the threshold for acceptable risk in an institution. Postoperative morbidity, categorized using Clavien–Dindo classification, is dependent on more than just intraoperative management.

Several alternative statistical techniques have been suggested in order to address the weaknesses of single outcome evaluation, among which cumulative sum (CUSUM) and other versions of it are the most popular ones. CUSUM method permits one to monitor the development of performance by calculating deviations from certain target values, thus providing the opportunity to detect inflection points characteristic of different stages of learning. More sophisticated variations of this approach include risk-adjusted CUSUM (RA-CUSUM) and learning curve CUSUM (LC-CUSUM) and try to take into account the differences caused by patient-related and procedure-related factors; however, all of these methods depend strongly on the choice of the target value and risk adjustment models [15,16].

Another weakness of the current techniques is the fact that process-based measures (operative time and intraoperative variables) are used quite often as indicators of surgeon’s overall competence. Such measures can reveal certain aspects of technical efficiency but do not take into account such important variables as oncologic adequacy; for instance, the status of margins and survival rate, as well as intraoperative decision-making. According to new data, efficiency is the first to improve followed by the rate of complications and oncologic results [17,18].

The main metrics currently used to assess learning curves in robotic HPB surgery, together with their principal strengths, limitations, and domains of assessment, are summarized in Table 1.

Anatomic difficulty must be described with the correct instrument: the Iwate difficulty score originates from the liver-resection difficulty model reported by Ban et al. [19], whereas the Southampton score developed by Halls et al. [12] and the Kawaguchi classification [20] are distinct systems. These scores should not be treated as synonyms. Furthermore, anatomic difficulty is not equivalent to patient-level risk, which may include age, BMI, frailty, comorbidity, prior abdominal surgery, hepatic function, and underlying parenchymal disease.

Overall, these findings indicate that the study of the learning curve in the context of robotic HPB surgery remains methodologically diverse and conceptually narrow. There is an urgent need to switch to multidimensional risk-adjusted frameworks of analysis.

## 6. Interpreting Cusum and Competency Thresholds

Conventional cumulative sum (CUSUM) plots often accumulate operative-time deviations from the mean of the same observed series. The location of a peak then depends on the case sequence, case-mix, and the chosen sample mean, which itself changes when more cases are added. Such a turning point is descriptive and does not establish a predefined, externally validated competency threshold. Learning-curve CUSUM (LC-CUSUM) instead tests performance against prospectively specified acceptable and unacceptable failure rates; risk-adjusted CUSUM (RA-CUSUM) incorporates an explicit expected-risk model for individual cases. LC-CUSUM is not inherently case-mix adjusted, and RA-CUSUM depends on the calibration and validity of its risk model. Both the endpoint and decision limits must be prespecified, and ideally validated externally.

The studies in Table 2 are a focused, non-exhaustive selection verified against accessible original publication records. Thresholds refer to the stated endpoint and should not be interpreted as universal competence standards.

## 7. Why Current Learning Curve Models May Be Misleading

While there are some common models for analyzing the learning curve for robot-assisted HPB surgery, these models have certain shortcomings that can affect the analysis and interpretation of the results obtained. The main problem with these models is related to the reliance on simple and one-dimensional indicators like operative time. Although all researchers report decreases in operative times, this is mostly an indicator of improvements in efficiency and not in surgical skills themselves. Therefore, identifying a plateau in operative time will give incorrect indications regarding the attainment of competence in other areas of performance [14,17].

The other critical issue with the learning curve is the insufficient consideration of risk adjustment and case mix. The initial stages of the learning curve involve an intentional selection of less complicated patients, which naturally results in better outcomes. With growing experience, the indications of the surgeons get extended to more complicated interventions. The growth in the difficulty level of cases may mask real gains in the skill level or vice versa. Research involving minimally invasive liver resection has shown that the learning curve may be affected significantly by the consideration of the case mix [7,20].

Moreover, selection and publication biases constitute significant though frequently overlooked problems. Available data come primarily from centers of high volume and well-trained environments with considerable prior experience with minimal access surgery, thus compromising external validity. Moreover, poor results observed during the early stages of adoption might be underrepresented, which leads to too positive an account of the learning curve. IDEAL framework for surgical innovations stresses the importance of consistent and structured reporting at each stage of adoption of a new technique; however, such reporting is far from being common in the realm of robotic HPB surgery [21,22].

Another problem is related to the perception of the linear and uniform progress throughout the learning curve. Actually, surgical education is a complex process affected by personal, technical, and organizational factors. Different performance areas—operating speed, complication rate, adequacy of resection—can change at their own pace and independently from each other. Research in surgical outcomes shows that there is no necessary connection between technical skills and reduction of complication rates [17,23].

Thirdly, the importance of non-technical and system-based issues, such as team experience, perioperative protocols, and institutional capabilities, is often neglected in current models. Robotic HPB surgery requires excellent performance of the team and high-level of institutional experience. Research dedicated to the study of the safety of surgeries and teamwork suggests that communication and coordination skills, as well as shared experience, have a significant effect on the results of perioperative period, and, thus, the positive changes might be attributed to team learning rather than only surgeon learning [24,25].

In summary, the presented discussion shows the need to interpret the results of learning curve models with caution. Although they give a good descriptive insight, they are unable to cover all aspects of the process of learning surgery, and, thus, more sophisticated models should be used to set realistic benchmarks.

## 8. Patient Risk Versus Procedural Complexity

Procedure-complexity scores and patient-level risk adjustment address related but distinct sources of heterogeneity. Liver difficulty scores consider anatomical and technical features, whereas patient-level models should consider, where clinically relevant, body mass index, comorbidity burden, prior abdominal surgery, cirrhosis, portal hypertension, liver function, neoadjuvant treatment, and baseline frailty. The IWATE criteria should not be conflated with the separate difficulty classifications developed by Halls and Kawaguchi. A complexity score alone does not control for patient risk. For pancreatic and biliary procedures, procedure-specific complexity and risk models are required rather than uncritical transfer of liver scores.

## 9. Toward a Multidimensional Framework for Learning Curve Assessment

The increasing awareness regarding the inherent shortcomings of conventional approaches to the construction of learning curves necessitates the development of an enhanced approach that could facilitate the evaluation of surgical learning in robotic HPB procedures. The current approach to the formation of a learning curve through the definition of a univariate measure based on a single inflection point does not take into account the complex nature of surgical performance and may oversimplify an inherently complex process. In this regard, learning should be viewed as a multidimensional and context-dependent process [9,26].

A multidimensional approach to the assessment of learning curves should be comprised of various dimensions of performance. The first dimension is related to technical efficiency and includes such measures as operative time, console time, and estimated blood loss that mainly depend on procedural familiarity, surgical dexterity, and workflow optimization. While these measures are highly responsive to early improvements and tend to experience significant shifts at the beginning of the learning process, they are of limited value when considered separately as they may not correlate with patient-based outcomes [10].

Second, the clinical outcome can be considered another vital area of the surgical outcome, consisting of postoperative morbidity, mortality, and failure-to-rescue rates. While efficiency-related indicators are determined solely by intraoperative parameters and change early in the learning curve, the above-mentioned parameters depend on intraoperative and perioperative variables and become stable later. Moreover, the complication rate can stay low at early stages owing to careful case selection, thereby concealing potential technical problems with surgery [17,18].

Third, in relation to HPB surgery specifically, oncological adequacy must be seen as an important area not sufficiently taken into account when developing learning curves. Indicators of surgical outcomes, such as R0 resection rate, lymph node retrieval, and long-term survival, are necessary to assess the quality of surgery; however, it takes time to obtain these results. Therefore, oncological endpoints are less likely to be included in learning curves. The omission of these indicators might result in incorrect evaluation of the learning process [27].

The fourth and least quantifiable domain relates to intraoperative decision-making and adaptability and pertains to the surgeon’s capacity to react to any unpredicted situations, change operative plans, and handle intraoperative complications. The latter dimension plays a vital role in surgery but still lacks measurement in the existing models. Progression in this sphere cannot be similar to that in technical efficiency and may strongly rely on experience, cognitive abilities, and previous exposure to difficult cases [26,28].

Besides the aforementioned key dimensions, an exhaustive approach should also encompass case complexity as a key contextual factor. Scoring systems of difficulty allow to divide cases into categories and give context to measures of performance. Learning curves become quite different when adjusted for case complexity, and improvement in the results can be caused not only by skill development but also by case selection [12,20].

It is worth noting that surgical learning occurs in an institutional context rather than in isolation from it. Robot-assisted HPB surgery involves high levels of complexity and the need for highly coordinated teams of specialists. Variables such as OR structure, anesthesia, nursing skills, and institutional volume play an important role in results and therefore can explain improvements not in terms of individual surgeon development but in the form of collective learning, which cannot be accounted for by traditional models of surgical learning [25,29].

With regard to methodology, the application of such a multidimensional approach necessitates the introduction of composite and longitudinal assessment methods. The identification of a threshold level is replaced by the simultaneous analysis of multiple performance parameters over time. Advanced statistical methods, like risk-adjustment or hierarchical modeling, can help include these parameters into account; however, they are not often applied in the literature [9,16].

Based on these considerations, we propose a multidimensional framework integrating technical, clinical, oncological, cognitive, complexity-related, team, and institutional domains to provide a more comprehensive assessment of learning in robotic HPB surgery (Table 3).

Under this conceptual model, the idea of a “learning curve” can be re-evaluated to adopt a more realistic perspective such as “learning landscape,” wherein various domains develop independently. It is possible that improvements in efficiency coexist with the variability of clinical results or oncologic performance, and advancement in one domain does not equate to being mature in other domains. This conceptualization gives rise to a more realistic understanding of surgical growth and development and could provide valuable insight into both the process of training and credentialing.

The utilization of a multidimensional model does not seek to negate the usefulness of current metrics but seeks rather to provide a richer context and incorporate the said metrics into an understanding of surgical learning.

## 10. Practical Implementation of the Proposed Framework

Existing instruments could serve as components rather than substitutes for a multidomain evaluation: OSATS and GOALS assess general technical performance, while GEARS (Global Evaluative Assessment of Robotic Skills) addresses robotic technical skills. Console-derived motion and instrument metrics may provide complementary information but require procedure-specific validation and should not be treated as surrogates for clinical safety. Intraoperative judgment could be assessed through blinded video review of decision points, structured assessment of response to unexpected findings, escalation and conversion decisions, and documented debriefing with multiple trained raters.

A preliminary implementation would use a domain-by-domain dashboard rather than a single unvalidated weighted total. Technical performance, patient safety, procedure-appropriate oncological quality, judgment, and team function would each receive predefined observable indicators. Safety-critical events should trigger case review irrespective of a favorable overall score; domains with inadequate evidence should be labeled “not yet assessable,” rather than passed. A “needs improvement” designation would prompt targeted supervision, simulation, and repeat assessment. No universal weighting, minimum score, or pass/fail threshold has been validated for robotic HPB surgery; these rules are proposals for prospective testing, not current credentialing standards.

The practical implementation of this framework would require institutional commitment and active engagement of relevant stakeholders, including HPB surgeons, trainees, anesthesiologists, operating room nurses, robotic program coordinators, hospital administrators, medical educators, and institutional quality and patient safety committees. Their involvement would help establish shared objectives, agree on clinically meaningful indicators, assign responsibilities, and promote acceptance of assessment as a tool for professional development rather than punishment. A multidisciplinary steering group could coordinate implementation, oversee evaluator training, and periodically review the framework in response to feedback. Institutional leadership support would be essential for integration into training pathways, clinical governance, and quality improvement programs.

Adequate resources are another prerequisite. Implementation would require protected assessment time, faculty development, simulation and proctoring opportunities, standardized data systems, and secure infrastructure for storing and analyzing performance data. Financial and organizational support would also be needed for video review, inter-rater calibration, longitudinal monitoring, and multidisciplinary feedback. These requirements may be more difficult to meet in lower-volume centers. A proportionate approach should therefore prioritize a minimum set of clinically relevant indicators before adding more complex domains. Interinstitutional collaboration could support shared training and common assessment standards.

A pragmatic implementation strategy could comprise three phases: initial stakeholder consultation, needs assessment, resource mapping, and agreement on procedure-specific indicators; pilot implementation with prospective data collection, structured feedback, and multidisciplinary review without immediate credentialing consequences; and subsequent evaluation of feasibility, acceptability, inter-rater reliability, cost, administrative burden, and associations with patient outcomes. Only after prospective validation and demonstration of sustainability should wider adoption or formal credentialing applications be considered.

## 11. Implications for Surgical Training and Credentialing

The awareness that surgical learning is a complex and non-linear phenomenon implies clear and inevitable consequences for surgical training programs and certification schemes in robotically assisted HPB surgery. In fact, the common practice of using procedural volumes as the only surrogate of competence seems insufficiently robust given the diversity, situationality, and contextual aspects of skills acquisition discussed above [9,26].

Essentially, this is the challenge to an implicit assumption in surgical training that experience can be reduced to procedural volumes alone. As a convenient and easily quantifiable criterion, procedure volume does not account for qualitative differences between cases in terms of complexity, decision-making during the operation, and ability to adapt. Outcome evidence suggests that surgeons with the same number of procedures performed display significant differences in complication rates and technical skills, revealing the discriminatory insufficiency of volume-based criteria [17].

In this light, it will become increasingly risky to base credentialing systems on quantitative requirements alone, and there will be a risk of misalignments between formal certification and actual competence. The consequences of misalignment of such types go beyond training equality issues; most importantly, there will be consequences for the safety of patients [8,26].

Such conceptual reframing can facilitate the move toward competence-based models of surgical training where advancement of students is based on demonstrating performance across several dimensions of competency. It needs to be highlighted that this approach does not negate the necessity to use quantitative measures; however, it requires them to be included in an overarching evaluation system. Evidence shows that objective measures (global rating scales, procedure-specific metrics) allow for more detailed evaluation of the skills of surgeons and are gaining validity in minimally invasive surgery [30,31]. However, their inclusion into surgical practice is heterogeneous and developing in robotic HPB surgery.

Another aspect of the implication is the need for structured and proficiency-based simulation training. In a multidimensional framework, simulation becomes not an addition to the process but the basic tool of acquiring skills prior to dealing with the clinical situation and progressing step-by-step through the necessary technical domains. The effectiveness of simulation is not limited by repetition but by the ability to receive feedback while practicing in a controlled manner, an approach proven to be more efficient [26,32]. Still, even a sophisticated simulation cannot duplicate the requirements of real-life surgery and, hence, has to be integrated into a broader training process.

Just as important is the reevaluation of mentoring and proctoring. Unlike the number of supervised procedures, mentoring and proctoring have to be personalized and competency-based. This approach is supported by the staged approach to surgical innovation suggested in the IDEAL framework [21]. It also takes into account the fact that the trajectories of learning processes are diverse and require personalized attention.

The inclusion of case complexity in training paths is yet another important step forward. The difficulty rating scale, especially when it comes to liver surgery, is an effective approach to differentiate the level of procedure and build a path of gradual autonomy. With the multidimensional understanding of competence, it is necessary to emphasize that success in low-complexity operations does not mean success in the complex ones. Without the incorporation of such a scale, training can go through premature advancement or too long supervised stage [20].

Several limitations should be considered when interpreting the evidence discussed in this narrative review. First, the literature on learning curves in robotic HPB surgery consists predominantly of retrospective observational studies, often from single high-volume institutions with considerable prior minimally invasive experience. This introduces selection bias and limits generalizability to lower-volume centers, newly established programs, and surgeons with different training backgrounds. The absence of contemporaneous controls and changes in perioperative practice over time complicate causal attribution of improved outcomes to surgical experience. Publication and reporting biases may further underrepresent unsuccessful early experiences.

Second, substantial methodological heterogeneity limits comparison between studies. Endpoints, definitions of proficiency, statistical approaches, and reported case-number milestones vary. Operative time, blood loss, conversion, and morbidity reflect not only surgeon skill but also patient selection, procedural complexity, anesthesia, and institutional workflow. Conventional CUSUM turning points depend on reference values, case order, and sample characteristics and do not establish validated competency thresholds. Risk-adjusted methods depend on complete covariate capture and well-calibrated models. Small cohorts and infrequent adverse events may further yield unstable estimates.

Third, previous open and laparoscopic experience, familiarity with robotic systems, dual-console access, mentoring, and team experience are inconsistently measured. Progressive selection of more challenging procedures creates time-dependent confounding, while substantial differences between liver, pancreatic, and biliary surgery limit transferability of thresholds. Long-term oncological outcomes, intraoperative judgment, non-technical skills, and team performance are rarely assessed together. Standardized, externally validated robotic HPB-specific instruments remain limited.

Finally, this review is narrative rather than systematic: its search and selection processes were not designed as an exhaustive, reproducible evidence synthesis, and no formal risk-of-bias assessment was undertaken. Some educational and methodological evidence comes from other surgical specialties and may not directly apply to HPB surgery. The proposed multidimensional framework remains conceptual and has not been prospectively validated. Its feasibility, reliability, responsiveness, predictive validity, optimal domain weighting, assessment frequency, resource requirements, and thresholds are unknown. It should not be interpreted as a validated credentialing instrument or a proven improvement over existing approaches. Prospective multicenter evaluation is required.

An additional challenge for future research is the continuing evolution of robotic surgical technology. The increasing availability of different platforms, instruments, imaging modalities, and integrated digital systems may influence both the acquisition and assessment of competence. Differences in console ergonomics, instrument articulation, visualization, feedback, and workflow may produce platform-specific learning trajectories, limiting transferability of thresholds between systems. Software and hardware upgrades may also alter performance independently of accumulated experience. Future studies should document platform, software version, instrumentation, and relevant technological changes to distinguish learning effects from technology-related effects.

At the same time, emerging technology may enable more objective and granular assessment. Artificial intelligence, computer vision, automated surgical phase recognition, instrument motion tracking, and robot-generated data could capture technical and workflow metrics not routinely available in clinical registries. AI-assisted video analysis may help characterize procedural steps and deviations from expected workflows [32,33]. However, data availability and comparability depend on platform capabilities, access to proprietary information, interoperability, and consistent metric definitions. Automated indicators must not be assumed to measure clinical competence without validation against meaningful patient outcomes.

Future research should therefore examine both the opportunities and complications introduced by technology-driven assessment. Multicenter studies across robotic platforms should identify shared performance domains while allowing appropriate technology-specific indicators. AI-based tools require external validation across centers, case complexity, and experience levels, with attention to algorithmic bias, reproducibility, transparency, data governance, and patient and professional privacy. Longitudinal studies should also determine whether new technologies create additional adaptation phases among experienced surgeons. Technological change should be treated as a dynamic contextual factor, not simply as an assessment instrument.

## 12. Limitations of the Evidence and the Proposed Model

This narrative synthesis is non-systematic and may omit relevant studies. The published evidence is dominated by selected centers and heterogeneous definitions of learning, and several educational or teamwork references derive from non-HPB specialties. Competency, proficiency, and mastery are useful conceptual distinctions but have no universally accepted operational thresholds in robotic HPB surgery. The multidimensional framework presented here has not been prospectively validated, its domain weights remain undefined, and superiority over conventional credentialing has not been demonstrated. Therefore, implications for policy should be interpreted as hypotheses and research priorities.

## 13. Future Directions and Research Agenda

In the move towards a multidimensional view of learning in robotic HPB surgery, there are some critical deficiencies that need to be addressed in order to bridge the gap between the current research and the real-world application of knowledge generated from the research. Despite increased acceptance of the fact that volume-based learning curves have their limitations, it is clear that there is still no standard set of tools used for multidimensional assessment of surgical skills [8,9].

One of the most important aspects of future research is developing multidimensional performance metrics that go beyond simple operative time and case volume measurements. Future research should focus on the development of evaluation tools that will include assessment of technical skill, intraoperative decision-making process, error management, and outcomes. This would allow for better assessment of competence and comparison between institutions and training methods. These metrics should be prospectively validated and outcome-based.

Future research should prospectively evaluate multidimensional, risk-adjusted assessment frameworks across institutions, robotic platforms, and procedural complexity levels. Successful implementation will require stakeholder engagement, adequate institutional resources, standardized data collection, and consideration of evolving surgical technologies. Until feasibility, reliability, and associations with meaningful clinical outcomes are established, these frameworks should be regarded as promising conceptual approaches rather than validated alternatives to existing training and credentialing standards.

A third important topic is the study of learning curves throughout time. Currently, most available literature includes retrospective studies and statistical models that do not consider the complexity and nonlinearity involved in acquiring new skills. Prospective multicenter studies with more sophisticated analytical methods such as mixed effects model and machine learning could be used to describe individual differences and to detect predictors of learning process [34].

The effect of case complexity and context also requires attention. Although some systems to assess complexity have been developed, the integration of these systems into learning frameworks is far from complete. Further research should be focused on studying the effect of complexity on outcomes when it comes to interaction with other variables such as surgeon experience, teamwork, etc. These data are necessary for designing safe and efficient learning programs, especially in risky fields like HPB surgery [20].

Also, it is necessary to broaden the view from individual expertise assessment to teamwork learning and organizational learning in order to understand better the process of acquiring the skills necessary for performing surgeries using robotic devices. In the field of HPB robotic surgery, the coordination of multidisciplinary processes plays an important role. However, the majority of studies are focused only on surgeons and do not involve team aspects [28].

The need for standardization in methodology and collaboration is another area where further research can be fruitful. Due to different types of studies being conducted using various measurements, there are limitations on how comparable the results can be and how easily evidence can be gathered. Standardized terminology and consensus on metrics can facilitate the process significantly [9].

## 14. Conclusions

In conclusion, this review emphasizes the inadequacy of the application of traditional volume-related models in capturing the concept of the learning curve in robotic hepatopancreatobiliary surgery. Available scientific literature clearly demonstrates that the phenomenon of surgical learning is a multifaceted, non-linear, and contextual, one which is determined by the interactions between technical and cognitive skills, complexity of cases, and the hospital setting.

From this point of view, procedural volume can be viewed as an imperfect and possibly misleading metric of competency, having serious consequences for training and credentialing. The transition from conventional volume-based measures of surgical performance to integrated and competency-based frameworks seems to be a promising but as yet unvalidated direction.

Future work should prospectively test structured, objective, and context-sensitive assessment frameworks against clinically meaningful outcomes. Until such validation is available, multidimensional assessment should be regarded as a promising proposal rather than a proven replacement for existing credentialing standards.

## Figures and Tables

**Table 1:** Metrics currently used to assess the learning curve in robotic HPB surgery.

Domain	Common Metrics	Main Advantages	Major Limitations	What It Actually Measures
Technical efficiency	Operative time; console time; docking time	Easy to collect; objective; sensitive to early improvements	Strongly influenced by team, workflow and case complexity	Procedural efficiency and familiarity
Blood loss	Estimated blood loss; transfusion rate	Clinically relevant; relatively easy to record	Influenced by tumor characteristics, vascular anatomy, liver quality and anesthetic management	Intraoperative performance and surgical difficulty
Conversion	Conversion to open surgery; conversion rate	Clinically meaningful; reflects difficulty and intraoperative management	Strongly affected by case selection and institutional threshold for conversion	Ability to manage minimally invasive surgery
Postoperative morbidity	Overall complications; Clavien–Dindo ≥ III; mortality; failure-to-rescue	Patient-centered; clinically relevant	Influenced by perioperative care, patient characteristics and case complexity	Clinical safety
Oncological outcomes	R0 rate; margin status; lymph-node retrieval; recurrence; survival	Directly relevant to oncological quality	Often require long follow-up; rarely incorporated into learning-curve models	Oncological adequacy
Statistical learning-curve models	CUSUM; LC-CUSUM; RA-CUSUM; risk-adjusted models	Can identify performance changes and inflection points	Highly dependent on target values, assumptions and risk-adjustment models	Temporal evolution of performance
Complexity adjustment	IWATE score; difficulty scores; risk stratification	Allows comparison across different case-mixes	Not universally applied; complexity itself may be incompletely captured	Contextualized surgical performance

Abbreviations: CUSUM, cumulative sum; LC-CUSUM, learning-curve cumulative sum; RA-CUSUM, risk-adjusted cumulative sum; IWATE, liver resection difficulty criteria.

**Table 2:** Selected verified robotic HPB learning-curve studies (focused narrative sample, not a systematic inventory).

Study	Procedure/*n*	Method	Endpoint	Reported Milestone	Interpretation/Limits
Rahimli et al., 2025 [3]	Robotic liver resection; 100	Operative-time trend and Iwate score; phase analysis	Operative efficiency and increasing complexity	Phases 1–20, 21–30, 31–65, 66–100; ~65 cases described for consistent proficiency	Single surgeon; rising complexity; phase labels are study-defined
Ratti et al., 2025 [6]	Robotic liver resection; 250-program cohort, 197 complexity-stratified cases	CUSUM; difficulty strata; two surgeons	Operative time and team efficiency	Low complexity ~15; intermediate ~10; high ~10 vs. ~18 by surgeon; team ~12	Threshold varies by surgeon and complexity; denominators differ
Boone et al., 2015 [4]	Robotic pancreatoduodenectomy; 200	Sequential outcome analysis	Blood loss; pancreatic fistula	Blood loss ~20; fistula ~40	Different endpoints yield different milestones; single-center experience
Shakir et al., 2015 [13]	Robotic distal pancreatectomy; 100	CUSUM and sequential outcomes	Operative time; readmission	Operative-time shifts ~20 and ~40; readmission improvement after ~40	High-volume center; endpoint-specific; nonrandomized

**Table 3:** A proposed multidimensional framework for assessing learning in robotic HPB surgery.

Domain	Core Variables	Stage of Learning	Potential Assessment Tools	Main Objective
Technical efficiency	Operative time; console time; docking time; EBL	Early	CUSUM; LC-CUSUM; longitudinal analysis	Assess procedural efficiency and robotic familiarity
Clinical outcomes	Overall morbidity; major morbidity; mortality; failure-to-rescue	Intermediate–late	Risk-adjusted models; CUSUM; hierarchical models	Assess patient safety
Oncological performance	R0 resection; margin status; lymph-node retrieval; recurrence; survival	Late	Longitudinal oncological analysis	Assess oncological adequacy
Intraoperative decision-making	Management of unexpected findings; intraoperative complications; change of operative strategy; conversion decisions	Intermediate–late	Structured assessment; expert evaluation; video-based assessment	Assess cognitive adaptability and judgement
Case complexity	IWATE/difficulty score; major vs. minor resection; tumor location; vascular involvement	Contextual factor	Risk stratification; complexity-adjusted models	Account for case-mix and avoid selection bias
Team performance	Communication; coordination; role distribution; workflow efficiency	Throughout learning	OTAS; structured team assessment	Distinguish individual from team learning
Institutional context	Robotic platform and technological infrastructure; annual robotic volume; institutional resources; structured training and proctoring; anesthesia and nursing experience; perioperative protocols	Throughout learning	Multilevel/hierarchical models	Account for organizational learning
Overall competency	Integration of technical, clinical, oncological and cognitive domains	Late	Composite/multidimensional assessment	Define competency beyond procedural volume

Note: Learning stages are illustrative, not validated milestones. Clinical safety and oncological appropriateness must be assessed from the beginning of practice; long-term outcomes become observable later and do not imply that competence is acquired only at that time. Abbreviations: EBL, estimated blood loss; CUSUM, cumulative sum; LC-CUSUM, learning-curve cumulative sum; OTAS, Observational Teamwork Assessment for Surgery; IWATE, liver resection difficulty criteria.

## Data Availability

No original datasets were generated or analyzed for this narrative review.
